# Use of Drugs With Risk of Heart Rate-Related Problems is Common in Norwegian Dementia Patients Treated With Acetylcholinesterase Inhibitors: A Prevalence Study Based on the Norwegian Prescription Database

**DOI:** 10.3389/fphar.2021.791578

**Published:** 2022-02-22

**Authors:** Anne Sverdrup Efjestad, Hege Ihle-Hansen, Vidar Hjellvik, Knut Engedal, Hege Salvesen Blix

**Affiliations:** ^1^ Hospital pharmacy Ahus, Nordbyhagen, Hospital Pharmacy Enterprices, South Eastern Norway, Oslo, Norway; ^2^ Department of Neurology, Oslo University Hospital, Oslo, Norway; ^3^ Department of Medicine, University of Oslo, Oslo, Norway; ^4^ Norwegian Institute of Public Health, Oslo, Norway; ^5^ Norwegian National Advisory Unit on Ageing and Health, Vestfold County Hospital, Oslo, Norway; ^6^ Department of Geriatrics, Oslo University Hospital, Oslo, Norway; ^7^ Department of Pharmacy, University of Oslo, Oslo, Norway

**Keywords:** acetylcholinesterase inhibitor, Alzheimer's disease, sex difference, pharmacoepidemiology of dementia, comorbidity in dementia, adverse drug reaction, prescription database

## Abstract

**Background:** Drugs commonly prescribed for heart rate control may induce adverse drug reactions in Alzheimer patients treated with acetylcholinesterase inhibitors (AChEIs). We have studied use of drugs with a known risk of Torsades de pointes (TdP) and drugs used to treat behavioral and psychological symptoms of dementia, as well as a combination of drugs with a known risk of TdP and drugs with a known heart rate-lowering effect, before and after initiating treatment with AChEIs.

**Methods:** The study applied data from the Norwegian Prescription Database for the period 2004–2016. Prescriptions of concomitant use of drugs in persistent users of AChEIs was studied in a follow-up period from 4 years before to 2 years after AChEI initiation in men and women of two age groups: 37–80 and 81–88 years.

**Results:** A small number of patients were prescribed haloperidol (∼1.5% The second year after AChEI initiation), digoxin/digitoxin (∼3%), and verapamil (∼1.3%), while a substantial proportion of the patients were prescribed betablockers (∼28%) and citalopram/escitalopram (∼17%). During follow-up, up to 6% of the study population were prescribed both betablockers and citalopram/citalopram in addition to AChEIs, a combination that increased over the follow-up period and was observed most frequently in women in the oldest age group.

**Conclusions:** A large proportion (∼44%) of patients treated with AChEIs were prescribed drugs that could cause bradycardic and prolonged time from the start of the Q wave to the end of the T wave (QT interval). Thus, action should be taken to reduce the combination of drugs with risk of bradycardia and prolonged QT interval. Medication review on a regular basis could be an option as an important risk-reducing intervention.

## Introduction

Acetylcholinesterase inhibitors (AChEIs) are usually prescribed early in the course of dementia due to Alzheimer’s disease (AD), and patients who respond well may take these drugs for several years (Hernandez et al., 2009). The target organ for AChEIs is the brain, but as the heart is also rich in cholinesterases, the AChEIs may adversely affect cardiac function due to the cholinergic effects, especially in older patients, resulting in risks of arrhythmias, prolonged time from the start of the Q wave to the end of the T wave (QT-interval), and Torsades de Pointes (TdP) (Isik et al., 2012). Of the AChEIs, only donepezil is classified with a known risk of QT prolongation and TdP (Crediblemeds, 2021). Still, the current knowledge on this topic is limited, and epidemiological studies to examine the comparative risk with use of the different AChEIs are missing (Huang and Alsabbagh, 2020). Effects on the heart is a class effect of the AChEIs (Editorial, 2006) and may result in bradycardia caused by blockage of cholinesterase connected to the vagal nerve, which can cause atrioventricular (AV) or sinoatrial block (Huang and Alsabbagh, 2020). The use of AChEIs has been associated with more than twofold risk of hospitalization due to bradycardia among elderly patients. In addition, those receiving concurrent therapy with negative chronotropic drugs, such as betablockers, digoxin, and verapamil, had an increased risk as well (Park-Wyllie et al., 2009). According to a US study among veterans with AD, patients taking betablockers, with earlier episodes of falls and of myocardial infarction, heart failure, or hypertension have been reported to be most vulnerable for a decrease in heart rate after initiating treatment with AChEIs (Hernandez et al., 2009).

Multiple medical comorbid diseases are common in people with dementia (Chen et al., 2017). Hypertension and cardiovascular disease, which are the most common preexisting comorbidities, are at the same time risk factors of dementia (Ruangritchankul et al., 2020). It is relevant for both sexes to reduce the cardiovascular risk factors to decrease the incidence of dementia (Chêne et al., 2015). As betablockers and cardiotropic calcium blockers are among the drugs that may increase heart-related problems like bradycardia and hypotension, and because bradycardia in older people is associated with syncope, arrhythmias, and falls, it is important to identify high-risk patients (Hernandez et al., 2009). The most serious adverse effects of digoxin are life-threatening cardiac disorders, and bradycardia is an early warning sign (Editorial, 2009). Through its heart-rate lowering effect, digoxin can cause TdP. The risk increases with coadministration of heart rate-lowering drugs and drugs that can prolong the QT interval, such as AChEIs (Editorial, 2009).

Behavioral and psychological symptoms of dementia (BPSD) are common in people with dementia and often treated with antidepressants and antipsychotics (Norwegian National guideline on dementia, 2021). The frequencies of atypical antipsychotic drugs for treatment of BPSD may be higher than for haloperidol. However, haloperidol was chosen in this study due to its known risk of QT interval prolongation. Among the commonly used agents for treatment of BPSD are escitalopram/citalopram, which also have a known risk of QT-interval prolongation and TdP, like donepezil (Crediblemeds, 2021). A combination of drugs with cardiac activity and AChEIs may explain harmful drug–drug interactions (Pasqualetti et al., 2015), likewise combinations of AChEIs with some psychotropic drugs. It is, therefore, of interest to study the prevalence of relevant drug combinations before and after initiating treatment with AChEIs.

Prescriptions of drugs like betablockers, digoxin, and verapamil can be considered as a proxy for heart disease, and patients with dementia suffering from preexisting cardiac disease, such as heart rate disturbances, are reported to have a higher risk of antipsychotic-induced arrhythmias and sudden cardiac death, related to prolongation of the QT interval (Bilotta et al., 2014).

Women will, in general, get cardiovascular disease 6–10 years later than men do and, explained by, different development of blood pressure between the sexes, partly due to women living longer than men (Hestad et al., 2020a). Adverse drug reactions (ADRs) are more common in women than in men, explained by factors such as higher drug levels as a result of pharmacokinetic and pharmacodynamic differences between the sexes and more polypharmacy in women than in men (Rochon et al., 2013; Lucas et al., 2016). The QT interval is generally longer in women, with a higher risk of drug-induced ventricular arrhythmias in women compared with men (Bilotta et al., 2014). Drugs that prolong the QT interval have a greater response in women than in men (Cubeddu, 2016).

Furthermore, age is of interest because aging is associated by pharmacokinetic changes, such as reduced renal excretion and hepatic clearance and altered sensitivity to drugs, such as cardiovascular and psychotropic drugs, and predisposes older persons to ADRs (Rosenthal and Nussinovitch, 2008), in particular, AD patients (Pasqualetti et al., 2015). Systolic blood pressure increases with age in patients with dementia disorders until about 80 years, after which it tends downward (Hestad et al., 2020b), and the association of elevated blood pressure with cognitive performance has been suggested to be different between the two sexes (Hestad et al., 2020a). A large proportion of older adults have complex arrhythmias, and prolongation of the QT interval is related to age-associated degenerative change in the conduction system (Isik et al., 2012).

On this background, the aim was to study changes, from 4 years before to 2 years after initiating treatment with AChEIs, of: 1) the prevalence of use of drugs commonly prescribed for heart rate control and drugs with a known risk of TdP commonly prescribed for treatment of BPSD, 2) the prevalence of combinations of drugs with a known risk of TdP and drugs with a known heart rate-lowering effect, and 3) the differences in prescription patterns of 1) and 2) by age and sex.

## Materials and methods

### Data

Drug use data were collected from the Norwegian Prescription Database (NorPD). The drugs are classified according to the Anatomical Therapeutic Chemical (ATC) ATC Classification Index with DDDs, 2017 Index with DDDs 2017. The data contain, for each drug dispensation, information about the patient [sex, year of birth, year and month of death (when relevant)] and the drug [date of dispensing, medicinal product name and formulation, ATC-code, number of defined daily doses (DDDs), and the number of tablets/capsules/plasters (allowing us to calculate treatment periods)]. For each dispensing, we had information on the number of tablets/capsules/plasters dispensed. We assumed a consumption of two tablets per day for rivastigmine (ATC code N06DA03) and one tablet per day for the other AChEIs. Drugs dispensed to patients in institutions, e.g., nursing homes, are not included in the NorPD.

We assumed that dispensed drugs were consumed. We had access to all of the above information between January 1, 2004 and December 31, 2016 for all persons being dispensed at least one prescription of an AChEI (ATC-code N06DA) in that period. In addition, the yearly total number of users of all prescribed drugs in the population by gender and 1-year age groups (up to and including 89 years) was available in the same period.

### Study population

The study population consisted of all persistent (see below) AChEI users living at home who—at an age of 88 years or younger—initiated AChEI treatment between January 1, 2008 and December 31, 2013. Those who died within 2 years after the year of AChEI treatment initiation, or who were not registered in the NorPD the second year after AChEI initiation (could be in nursing home), were excluded. They were followed from 1,460 days (4 years) before initiation to 729 days (2 years) after initiation. For the general population, data with 1-year age resolution was only available up to, and including, 89 years; hence, the age limit of 88 was set for the study population to make a comparison with the general population possible. Due to the aging process and increased comorbidities, the study population was stratified into two subgroups according to age over and under 80. We used the day of treatment initiation as a surrogate marker of the time of the AD diagnosis (Norwegian National guideline on Dementia, 2021).

A 1-year washout period is commonly used for defining incident drug use. Here, incident drug use was defined as being prescribed an AChEI drug after more than 365 successive days with no AChEI prescriptions, as some patients had several AChEI use periods separated by 365 days without prescriptions. For these, follow-up started at the date of the prescription initiating the longest AChEI use period (index date). Treatment length was computed as the number of days from index date until the drug dispensed in the last prescription of the longest AChEI use period was supposed to be consumed. The earliest possible index date was January 1, 2005. We only included patients with index date between January 1, 2008 and December 31, 2013 to capture drug use 4 years before and 2 years after initiation.

### Incident and Persistent Use of Acetylcholinesterase Inhibitors

Incident use was defined as being prescribed an AChEI drug after 365 successive days with no AChEI prescriptions. For patients with incident use more than once (several 365-day periods without AChEI prescriptions), follow-up started at the date initiating the AChEI period with the largest number of subsequent AChEI prescriptions <365 days apart. Treatment with AChEI should be evaluated 90–180 days after initiation according to guidelines; therefore, we considered, due to a possible delay, users who continued treatment 8 months (240 days) after initiation as persistent users (Efjestad et al., 2019). Thus, an incident user was defined as persistent if any of the following was true (Sverdrup Efjestad et al., 2017): 1) A new prescription was given between day 210 and day 240 after initiation, 2) drugs for at least 210-day consumption were prescribed during the first 210 days from initiation, or 3) the last prescription before day 210 lasted to day 210.

### Sex Differences and Comparison With Drug Use in the General Population

The use of, e.g., betablockers in the study population the first year after initiation of AChEI was compared with the use in the general population as follows: For each of the years 2008–2013, the age-adjusted prevalence of betablocker use in the general population was computed gender-wise, using the age distribution of those initiating AChEI, the actual year as reference. The overall age-adjusted prevalence in the general population was thereafter estimated as $\sum_{y=2008}^{2013}u_{y}/\sum_{y=2008}^{2013}N_{y}$ , where *u*_*y*_ and *N*_*y*_ are the age-adjusted number of betablocker users and the population in year *y*, respectively. In the study population, we are able to follow the same individual over a 6-year period. This is not possible in the general population. When comparing the betablocker use in AChEI initiators X years before/after initiation with the general population, the dispensing years and the age in the general population was shifted X years. As an example, betablocker use in the 81–88 years age group 4 years before initiation was compared with use in the 77- to 84-year-old persons (as the age group was 4 years younger than the 81–88 group 4 years before, i.e., 77- to 84-year olds) in 2004–2009 in the general population. Betablocker use in the same age group the second year after initiation was compared with use in the 82- to 89-year olds in 2009–2014 in the general population. Age-adjusted prevalence ratios (PRs) for each gender were calculated by dividing the prevalence in the AChEI users by the age-adjusted prevalence in the general population.

The proportions of the study population and the general population receiving at least one prescription of haloperidol, citalopram/escitalopram, verapamil, betablockers, diuretics, and digitoxin/digoxin the 4 years before initiation of AChEI and the 2 years after were studied. Digitoxin has been the preferred digitalis drug in Norway, but was replaced with digoxin following a period with delivery problems in 2011. The two generics have about the same pharmacological profile (Haga et al., 2016).

### Drugs or Drug Groups

Prevalence of haloperidol (ATC code N05AD01), citalopram (N06AB10) or escitalopram (N06AB04), verapamil (C08DA01), betablockers (C07), and digitoxin/digoxin (C01A) was studied in the 4 years before and the 2 years after initiation of AChEIs (prevalence of use in each of the six 365-day periods during the 6 years of follow-up).

In addition, we studied prevalence of concomitant use of betablockers + citalopram or escitalopram, and of verapamil + citalopram or escitalopram in the 4 years before and the 2 years after initiation of AChEIs. Citalopram/escitalopram were selected as drugs with known risk of TdP, commonly used in the treatment of BPSD.

### Statistical Analysis

R versions 3.1.0–4.0.2 (R Core Team, 2014) were applied for descriptive statistics; proportions with 95% confidence intervals (CIs) were computed for the study population. The CIs were computed using the “binom.confint” function in the “binom” package in R with method = “wilson.” Age-adjusted prevalence rates were computed for the general population with the study population as reference, using the “ageadjust direct” function in the “epitools” package in R. The CIs for the general population are very narrow and, hence, not shown.

### Ethics and Data Protection Regulations

All the pharmacies in Norway register prescriptions electronically, and the information is sent in monthly reports to NorPD. The personal ID number of the patient and the ID numbers of the prescribers are replaced by a unique pseudonym by Statistics Norway. This makes it possible to link drug use to individuals without knowing their identity. Personal information is not disclosed from the NorPD. The database is governed by the national regulation of October 17, 2003 about the collection and processing of health data in the Norwegian Prescription Database. The NorPD generated pseudonymous files for research purposes, as regulated by Norwegian law for health register (FOR-2003-10-17-1246, 2003); hence, there was no demand of additional approval by the ethics committee. According to the national regulations for the NorPD at the study start, approval by an ethical committee was only necessary for studies where NorPD was linked to other registers. No other registers are involved in this study. Thus, no ethical committee has been contacted for this study. The data holder of NorPD, the Norwegian Institute of Public Health, has approved a full Data Protection Impact Assessment (DPIA) of the project.

## Results

**FIGURE 1:**
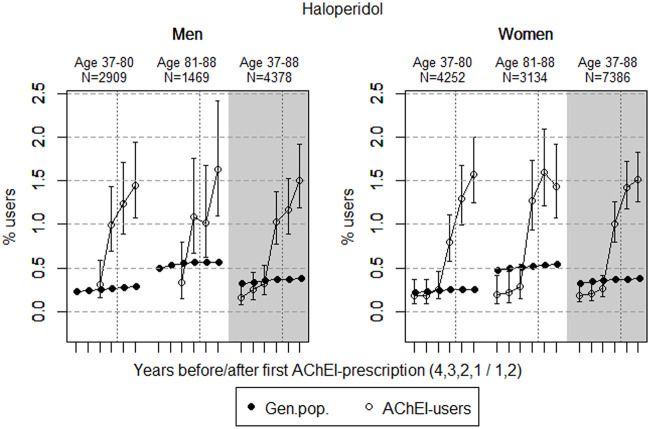
Open circles: Proportion of men and women, respectively, of the study population who filled at least one prescription of haloperidol (ATC N05AD01) in the 4 years before acetylcholinesterase inhibitors (AChEI) initiation and the 2 years after with 95% confidence intervals. Years with less than five users in one or both of the age groups are not shown. Bullets: The corresponding age adjusted proportion in the general population. Dashed vertical lines indicate AChEI initiation. The size of each age group in the study population is given on top.

**FIGURE 2:**
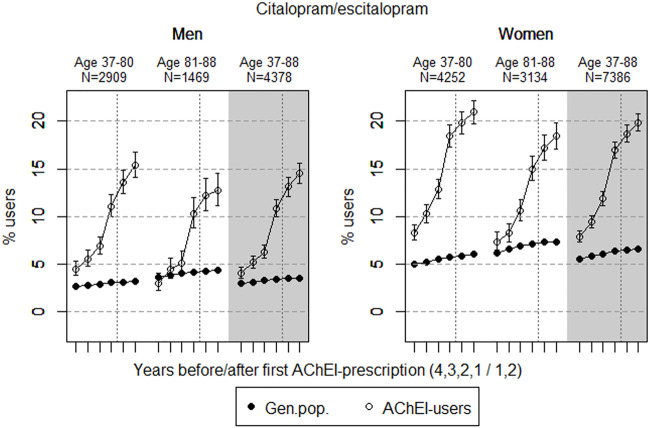
Open circles: Proportion of men and women, respectively, of the study population who filled at least one prescription of citalopram or escitalopram (ATC N06AB04 or 10) in the 4 years before AChEI initiation and the 2 years after with 95% confidence intervals. Bullets: The corresponding age adjusted proportion in the general population. Dashed vertical lines indicate AChEI initiation. The size of each age group in the study population is given on top.

**FIGURE 3:**
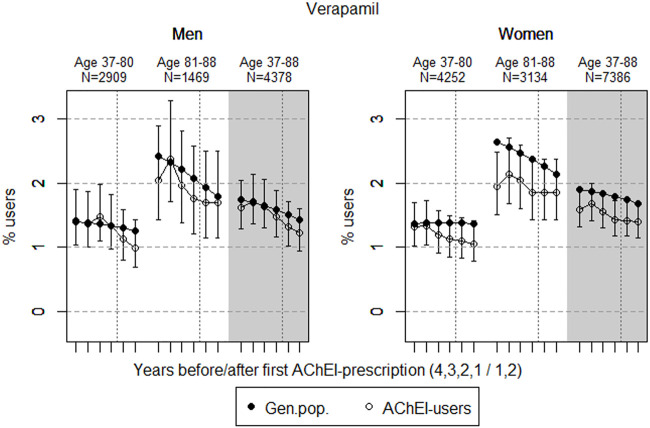
Open circles: Proportion of men and women, respectively, of the study population who filled at least one prescription of verapamil (ATC C08DA01) in the 4 years before AChEI initiation and the 2 years after with 95% confidence intervals. Bullets: The corresponding age adjusted proportion in the general population. Dashed vertical lines indicate AChEI initiation. The size of each age group in the study population is given on top.

**FIGURE 4:**
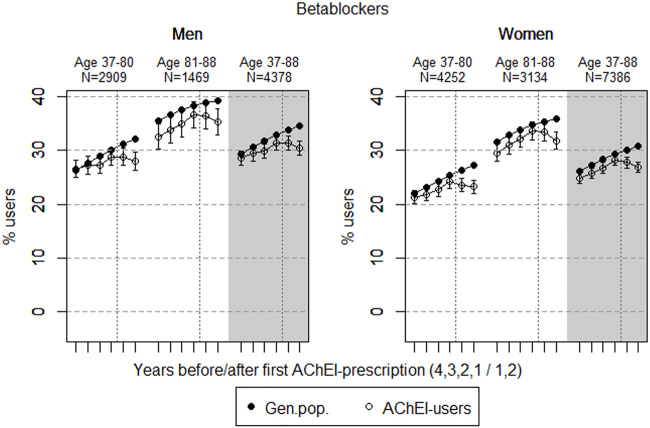
Open circles: Proportion of men and women, respectively, of the study population who filled at least one prescription of betablockers (ATC C07) in the 4 years before AChEI initiation and the 2 years after with 95% confidence intervals. Bullets: The corresponding age adjusted proportion in the general population. Dashed vertical lines indicate AChEI initiation. The size of each age group in the study population is given on top.

**FIGURE 5:**
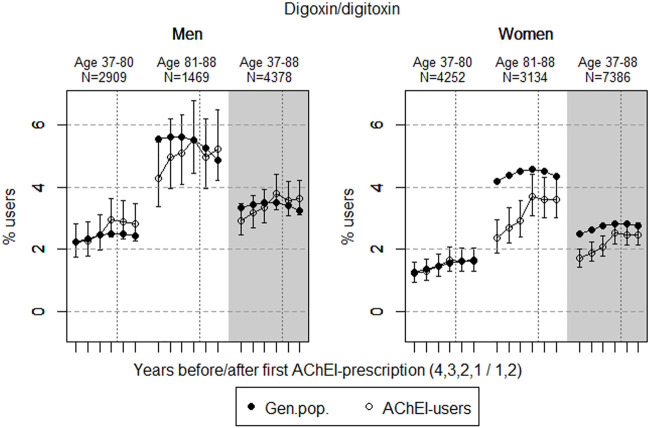
Open circles: Proportion of men and women, respectively, of the study population who filled at least one prescription of digoxin or digitoxin (ATC C01A) in the 4 years before AChEI initiation and the 2 years after with 95% confidence intervals. Bullets: The corresponding age adjusted proportion in the general population. Dashed vertical lines indicate AChEI initiation. The size of each age group in the study population is given on top.

**FIGURE 6:**
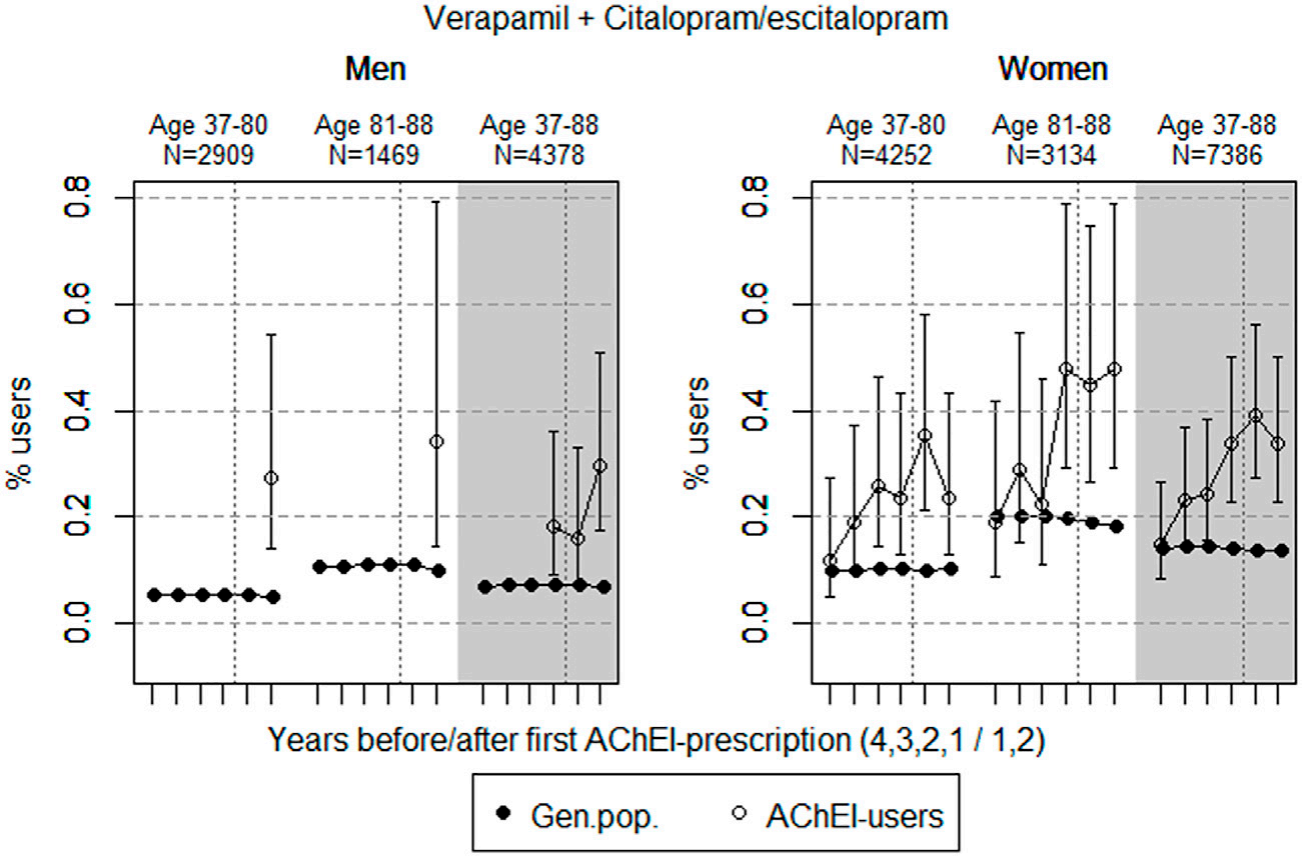
Open circles: Proportion of men and women, respectively, of the study population who used combinations of verapamil and citalopram/escitalopram in the 4 years before AChEI initiation and the 2 years after with 95% confidence intervals. Years with less than five users in one or both of the age groups are not shown. Bullets: The corresponding age adjusted proportion in the general population. Dashed vertical lines indicate AChEI initiation. The size of each age group in the study population is given on top.

**FIGURE 7:**
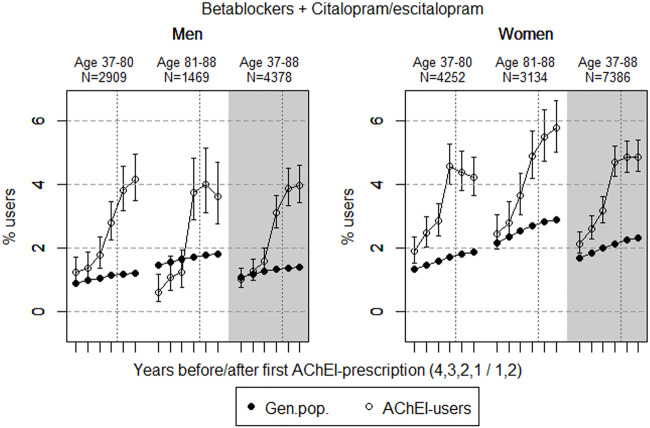
Bullets: Proportion of men and women, respectively, of the study population who used combinations of betablockers and citalopram/escitalopram in the 4 years before AChEI initiation and the 2 years after with 95% confidence intervals. Circles: The corresponding age adjusted proportion in the general population. Dashed vertical lines indicate AChEI initiation. The size of each age group in the study population is given on top.

The study population consisted of 11.764 persistent AChEI users aged 37–88 years who initiated treatment between January 1, 2008 and December 31, 2013. The percentage of women was 59% in the 37–80 age group and 68% in the 81–88 age group, in all 63%. The proportion of the study population receiving at least one prescription of haloperidol, citalopram/escitalopram, verapamil, betablockers, and digitoxin/digoxin the 4 years before initiation of AChEI and the 2 years after, are shown for men and women separately in Figures 1–5, respectively. The age-adjusted proportion of men and women in the general population receiving the same drugs are, for comparison, shown in the same figures. Of the men and women in the study population, 43.4% and 43.9%, respectively, received a prescription in at least one of the five drug groups the second year after AChEI initiation (Supplementary Figure S1; Supplementary Table S1). The corresponding proportions in the age-adjusted general population were 38.6% and 36.9%. Proportions of men and women of the two age groups of the study population who used combinations of verapamil and citalopram/escitalopram and of betablockers and citalopram/escitalopram, respectively, are shown in Figures 6, 7, in addition to the age-adjusted proportion of men and women in the general population receiving the same combinations.

We found no differences between the two age groups related to prescription of the various AChEIs. As donepezil was the first AChEI on the market, between 74.0% (youngest age group) and 75.2% (oldest age group) used this drug.

### Prevalence of Use in the General Population

A change in prevalence in the general population over the 6 years of follow-up can mainly be explained by the 6 years increase in age. Women of the general population showed a slightly higher prevalence of use of citalopram/escitalopram than men. Men of the general population showed a slightly higher prevalence of use of digitoxin/digoxin and betablockers than women. A notable age-related increase in the use of verapamil, betablockers, and digoxin/digitoxin was observed in both sexes. The change during follow-up was small: In the 37- to 88-year age group, the prevalence for *verapamil* decreased from 1.7% to 1.4% in men and from 1.9% to 1.7% in women; for *betablockers*, it increased from 29.4% to 34.6% in men and from 26.1% to 30.9% in women; for *digoxin/digitoxin*, it decreased from 3.4% to 3.3% in men and increased from 2.5% to 2.8% in women. For *haloperidol* and *citalopram/escitalopram*, no specific sex differences were observed during the study period. For all drugs, the pattern of change was the same during the study period in the different age groups and between the two sexes. Age-specific proportions (37–80 and 81–88 years) are shown in Table 1 and Supplementary Table S1.

**TABLE 1:** Prevalences of use of the studied drugs during following-up time by age and sex.

Years before (−) and after (+) AChEI initiation		Ages 37–80						Ages 81–88					
		Men			Women			Men			Women		
		−4 year	−1 year	+2 year	−4 year	−1 year	+2 year	−4 year	−1 year	+2 year	−4 year	−1 year	+2 year
Haloperidol	Prop SP	0.17	1.00	1.44	0.19	0.80	1.58	0.14	1.09	1.63	0.19	1.28	1.44
	Prop GP	0.23	0.27	0.29	0.22	0.25	0.26	0.50	0.57	0.57	0.47	0.52	0.54
	Ratio SP/GP	0.75	3.80	5.10	0.87	3.20	6.20	0.27	1.91	2.89	0.41	2.44	2.64
Citalopram/escitalopram	Prop SP	4.5	11.1	15.4	8.3	18.4	21.0	3.0	10.3	12.7	7.4	15.0	18.4
	Prop GP	2.6	3.0	3.2	4.9	5.7	6.0	3.6	4.2	4.3	6.2	7.1	7.3
	Ratio SP/GP	1.72	3.7	4.8	1.68	3.2	3.5	0.82	2.46	2.96	1.19	2.11	2.51
Verapamil	Prop SP	1.41	1.34	1.00	1.32	1.13	1.06	2.04	1.77	1.70	1.95	1.85	1.85
	Prop GP	1.40	1.34	1.27	1.37	1.39	1.36	2.43	2.08	1.79	2.64	2.37	2.14
	Ratio SP/GP	1.01	1.00	0.79	0.96	0.81	0.78	0.84	0.85	0.95	0.74	0.78	0.86
Betablockers	Prop SP	26.5	28.8	27.9	21.2	24.2	23.2	32.5	36.6	35.3	29.5	33.6	31.8
	Prop GP	26.3	30.1	32.2	21.9	25.3	27.2	35.4	38.3	39.3	31.6	34.7	35.8
	Ratio SP/GP	1.01	0.96	0.87	0.97	0.96	0.85	0.92	0.96	0.9	0.93	0.97	0.89
Digoxin/digitoxin	Prop SP	2.23	2.96	2.82	1.22	1.65	1.62	4.30	5.50	5.20	2.36	3.70	3.60
	Prop GP	2.24	2.51	2.44	1.25	1.57	1.65	5.60	5.50	4.90	4.20	4.60	4.30
	Ratio SP/GP	1.00	1.18	1.15	0.98	1.05	0.98	0.77	1.00	1.08	0.56	0.81	0.83

Note. Proportion (%) of men and women, respectively, of the study population (SP) who filled at least one prescription of the studied drugs in the fourth and first years before AChEI initiation and the second year after, and corresponding proportion in the age adjusted general population (GP), and prevalence ratios between SP and GP. Confidence intervals are given in Supplementary Table S1.

### Prevalence of Use in the Study Population

Women of the study population showed a higher prevalence of use of citalopram/escitalopram and verapamil than men. A notable age-related increase in the use of betablockers and digoxin/digitoxin was observed. During the study period, the prevalence of use in the study population overall (age 37–88) compared with the general population was higher for haloperidol and citalopram/escitalopram, but lower for verapamil, betablockers, and digoxin/digitoxin in women. There were some age group-specific exceptions to this pattern, though, see details for the different drug groups below and in Table 1 and Supplementary Table S1.

### Haloperidol

The prevalence of use of haloperidol strongly increased in both genders from 2 years prior to introduction of AChEIs in both sexes (Figure 1); however, it slightly decreased in the 81–88 age group in women in the last year of the study period. Regarding the use in men, the 37- to 80-year group increased from <0.3% in the fourth year before initiation to 1.4% (1.1%–1.9%) in the second year after, and the corresponding increase in the 81- to 88-year old was from <0.3% in the fourth year before initiation to 1.6% (1.1%–2.4%) in the second year after (exact numbers not shown due to <5 users in one or both of the age groups). Regarding the use in women, the 37- to 80-year group increased from 0.2% (95% CI: 0.1%–0.4%) in the fourth year before initiation to 1.6% (1.2%–2.0%) in the second year after, and the corresponding increase in the 81- to 88-year old was from 0.2% (95% CI: 0.1%–0.4%) in the fourth year before initiation to 1.4% (1.1%–1.9%) in the second year after. Compared with the general population, the prevalence ratio (PR) in men increased from <1 to 5.1 over the 6 years in the 37- to 80-year old and from <1 to 2.9 in the 81- to 88-year old. In women, the PR increased from 0.9 to 6.2 over the 6 years in the 37- to 80-year old and from 0.4 to 2.6 in the 81- to 88-year old.

### Citalopram and Escitalopram

The prevalence of use of citalopram and escitalopram strongly increased in both sexes over the 6-years interval and was higher in women than in men in the two age groups (Figure 2). Regarding the use in men, the 37- to 80-year group increased from 4.5% (95% CI: 3.8%–5.3%) in the fourth year before initiation to 15.4% (14.1%–16.7%) in the second year after, and the corresponding increase in the 81- 88-year old was from 3.0% (95% CI: 2.2%–4.0%) in the fourth year before initiation to 12.7% (11.1%–14.5%) in the second year after. Regarding the use in women, the 37- to 80-year group increased from 8.3% (95% CI: 7.5%–9.1%) in the fourth year before initiation to 21.0% (19.8%–22.2%) in the second year after, and the corresponding increase in the 81- to 88-year old was from 7.4% (95% CI: 6.5%–8.3%) in the fourth year before initiation to 18.4% (17.1%–19.8%) in the second year after. Compared with the general population, the PR in men increased from 1.7 to 4.8 over the 6 years in the 37- to 80-year old and from 0.8 to 3.0 in the 81- to 88-year old. In women, the PR increased from 1.7 to 3.5 over the 6 years in the 37- to 80-year old and from 1.2 to 2.5 in the 81- to 88-year old.

### Verapamil

The prevalence of use of verapamil slightly decreased prior to introduction of AChEIs in both sexes (Figure 3). The use in men in the 37 to 80-year group decreased from 1.4% (95% CI: 1.0%–1.9%) in the fourth year before initiation to 1.0% (0.7%–1.4%) in the second year after, and the corresponding decrease in the 81- to 88-year old was from 2.0% (95% CI: 1.4%–2.9%) in the fourth year before initiation to 1.7% (1.2%–2.5%) in the second year after. Regarding the use in women, the 37- to 80-year group decreased from 1.3% (95% CI: 1.0%–1.7%) in the fourth year before initiation to 1.1% (0.8%–1.4%) in the second year after, and the corresponding decrease in the 81- to 88-year old was from 1.9% (95% CI: 1.5%–2.5%) in the fourth year before initiation to 1.9% (1.4%–2.4%) in the second year after. Compared with the general population, the PR decreased in men from 1.01 to 0.79 over the 6 years in the 37- to 80-year old and increased from 0.84 to 0.95 in the 81- to 88-year old. In women, the PR decreased from 0.96 to 0.78 over the 6 years in the 37- to 80-year old and increased from 0.74 to 0.86 in the 81- to 88-year old.

### Betablockers

The prevalence of use of betablockers slightly increased in both sexes from 4 years prior to introduction of AChEIs (Figure 4), more or less in parallel with the general population, but decreased in both sexes from 1 year prior to introduction of AChEIs. Regarding the use in men, the 37- to 80-year group increased from 26.5% (95% CI: 24.9%–28.1%) in the fourth year before initiation to 28.8% (27.2%–30.5%) 1 year before, and thereafter, it decreased to 27.9% (26.3%–29.6%) in the second year after. The corresponding increase in the 81- to 88-year old was from 32.5% (95% CI: 30.2%–35.0%) in the fourth year before initiation, *via* 36.6% (34.2%–39.1%) 1 year before, to 35.3% (32.9%–37.7%) in the second year after. Regarding the use in women, the 37- to 80-year group increased from 21.2% (95% CI: 20.0%–22.5%) in the fourth year before initiation to 24.4% (23.0%–25.5%) 1 year before, and thereafter, it decreased to 23.2% (22.0%–24.5%) in the second year after. The corresponding increase in the 81- to 88-year old was from 29.5% (95% CI: 27.9%–31.1%) in the fourth year before initiation, via 33.6% (32.0%–35.3%) 1 year before, to 31.8% (30.2%–33.5%) in the second year after. Compared with the general population, the PR decreased in men from 1.01 to 0.87 over the 6 years in the 37- to 80-year old and from 0.92 to 0.90 in the 81- to 88-year old. In women, the PR decreased from 0.97 to 0.85 over the 6 years in the 37- to 80-year old and from 0.93 to 0.89 in the 81- to 88-year old.

### Digoxin/Digitoxin

The prevalence of use of digoxin/digitoxin slightly increased in both sexes in the study population overall (Figure 5), and the prevalence of use was higher in men than in women. The use in men in the 37- to 80-year group increased from 2.2% (95% CI: 1.8%–2.8%) in the fourth year before initiation to 2.8% (2.3%–3.5%) in the second year after. The overall increase in the 81- to 88-year old was from 4.3% (95% CI: 3.4%–5.4%) in the fourth year before initiation to 5.2% (4.2%–6.5%) in the second year after. The use in women in the 37- to 80-year group increased from 1.2% (95% CI: 0.9%–1.6%) in the fourth year before initiation to 1.6% (1.3%–2.0%) in the second year after, and the corresponding increase in the 81- to 88-year old was from 2.4% (95% CI: 1.9%–3.0%) in the fourth year before initiation, flattening out from 1 year before to 3.6% (3.0%–4.3%) in the second year after. Compared with the general population, the PR in men increased from 1.00 to 1.15 over the 6 years in the 37- to 80-year old and from 0.77 to 1.08 in the 81- to 88-year old. In women, the PR was stable at 0.98 over the 6 years in the 37- to 80-year old and increased from 0.56 to 0.83 in the 81- to 88-year old.

### Combinations of Verapamil and Citalopram/Escitalopram

The proportion of the study population overall using combinations of verapamil and citalopram/escitalopram was very low, particularly in men (Figure 6). The use in men in the study population overall increased from <0.1% in the fourth year before initiation (<5 users) to 0.3% (0.2%–0.5%) in the second year after. The use in women in the study population overall increased from 0.1% (95% CI: 0.1%–0.3%) in the fourth year before initiation to 0.3% (0.2–0.5%) in the second year after. Compared with the general population, the PR increased in men from <1 to 4.4 over the 6 years in the 37- to 88-year old. In women, the PR increased from 1.1 to 2.5 in the 37- to 88-year old over the 6-year interval.

### Combinations of Betablockers and Citalopram/Escitalopram

The proportion of the study population using combinations of betablockers and citalopram/escitalopram increased over the study period in the age group 37–88 and was higher in women than in men (Figure 7). The use in men in the study population overall increased from 1.0% (95% CI: 0.8%–1.7%) in the fourth year before initiation *via* 3.1 (2.6%–3.7%) in the first year before to 4.0% (3.4%–4.6%) in the second year after. The use in women in the study population overall increased from 2.1% (95% CI: 1.8%–2.4%) in the fourth year before initiation to 4.7% (4.3%–5.2%) in the first year before, and thereafter, it flattened out to 4.9% (4.4–5.4%) in the second year after. For the use in men, the 37- to 80-years group increased from 1.2% (95% CI: 0.9%–1.7%) in the fourth year before initiation to 4.2% (3.5%–4.9%) in the second year after. For the use in men, the 81- to 88-year group increased from 0.6% (95% CI: 0.3%–1.2%) in the fourth year before initiation to 3.7% (2.9%–4.8%) in the first year before and 3.6% (2.8%–4.7%) in the second year after. For the use in women, the 37- to 80-years group increased from 1.9% (95% CI: 1.5%–2.4%) in the fourth year before initiation to 4.6% (4.0%–5.3%) in the first year before, and thereafter, it decreased to 4.2% (3.6–4.9%) in the second year after. The corresponding increase in the 81- to 88-year old was from 2.5% (95% CI: 2.0%–3.1%) in the fourth year before initiation to 4.9% (4.2%–5.7%) in the first year before initiation and 5.8% (95% CI: 5.0%–6.6%) in the second year after. Compared with the general population, the PR increased in men from 1.0 to 2.8 over the 6 years in the 37- to 88-year old. In women, the PR increased from 1.3 to 2.1 in the 37- to 88-year old over the 6-year interval.

## Discussion

In users of AChEIs, a high prevalence of use of betablockers and of citalopram/escitalopram was found. The prevalence of use of haloperidol and citalopram/escitalopram was higher in the study population compared with the general population. The prevalence of use of verapamil was low in the general population and even lower in the study population, and use of the digitalis drugs was flattening out in the study population following introduction of AChEIs. The proportion of the study population with concomitant use of betablockers and citalopram/escitalopram was higher in women than in men. We have searched the literature, and unfortunately, we could not find any comparative studies.

### Haloperidol

Haloperidol has been shown to be used with a higher frequency in patients having a significantly greater risk of QT prolongation and TdP (older, medically ill, hospitalized patients) than other antipsychotics (Beach et al., 2018). The overall low prevalence of use of haloperidol reflects the restricted use of typical antipsychotics in dementia. However, the prevalence of use in the study population was higher than in the general population, and the use is not in accordance with the Norwegian National guideline on dementia, 2021. The increase in both sexes from 2 years prior to the dementia diagnosis may reflect an increase in the severity of the BPSD symptoms during the course of the AD.

### Citalopram and Escitalopram

The strong increase in the prevalence of use of citalopram and escitalopram in both sexes over the 6-year interval may indicate that incidence of depression has increased, or that symptoms of depression are more often treated with an antidepressant, especially in women. It could also be that these drugs were used to treat other BPSDs due to the restricted use of antipsychotics. The prescription pattern was not markedly influenced by initiation of AChEIs. Citalopram has, together with warfarin, shown to be the most frequently observed substance causing clinically relevant drug–drug interactions in hospitalized people with dementia, partly explained by the prolongation of the QT interval not seen among other selective serotonin reuptake inhibitors (SSRIs) (Sönnerstam et al., 2018). The high prevalence of use is therefore a concern, especially in women being more sensitive to ADRs. Norwegian National guideline on dementia, (2021) recommend only a restricted use of SSRIs in AD. According to these guidelines, an SSRI drug should be offered as additional treatment of depression, not of BPSD in general, and only when appropriate environmental psychological and/or psychotherapeutic measures have been attempted without achieving the desired effect.

### Verapamil

The low prevalence of use of verapamil in the general population, which is even lower in the study population, reflects that this class IV antiarrhythmic drug is not the first drug of choice in treating heart frequency problems. Patients taking verapamil must be carefully monitored with regard to adverse effects like bradycardia, and interactions may be enhanced by verapamil being an inhibitor of p-glycoprotein. As an example, we can expect a much higher bioavailability of digoxin when accompanied by verapamil. The decrease in prevalence of use in both sexes of the study population from 2 to 3 years before the AD diagnosis indicates that the drug is prescribed with caution, possibly because of the known risk profile and/or ADRs.

### Betablockers

The increase in the prevalence of the use of betablockers probably reflects the increase in cardiovascular disease among older people, especially in older people with dementia, as shown in the 81- to 88-year old in both sexes. From the aspect of risks, even the smallest doses of betablockers may induce severe bradycardia and disorders of AV conduction in the more sensitive elderly patients, which can clinically be manifested as vertigo and falls with possible serious injury. Such effects may be enhanced by the administration of other medication with effects on the heart (Kubesova et al., 2013), and thereby explain the reduction in the prevalence of use of betablockers in both sexes and age groups after AChEI initiation.

### Digoxin/Digitoxin

Digoxin is a cardiac glycoside that is commonly used among older adults in the treatment of congestive heart failure and atrial fibrillation. However, since AChEIs and digoxin can interact to give changes in the heart rate or cardiac conduction, co-prescription of digoxin with AChEIs have the potential to increase the risk of ADRs (Bentué-Ferrer et al., 2003). The potentially life-threatening cardiac adverse effects of digoxin like bradycardia could easily occur due to the narrow therapeutic window of digoxin (Editorial, 2009). The prevalence of use of the digitalis drugs was flattening out in the study population following introduction of AChEIs, especially in the older age group in women, which indicates a restricted use in people with dementia. This could be explained by ADRs, especially in older women, and fewer patients with indications for use of these drugs.

### Drug combinations

The small but higher proportion of the combination of verapamil and citalopram/escitalopram in the study population compared with the general population, especially in the higher age group following initiation of AChEIs, possibly reflects the high and increasing prevalence of use of citalopram/escitalopram in the study population.

The proportion of the study population with concomitant use of betablockers and citalopram/escitalopram was higher in women than in men. A reduced prevalence was observed in women in the 37- to 80-year old group, while the prevalence of use seemed to continue to increase in men. However, in the oldest age group, the prevalence of use declined in men following initiation of AChEIs, while it increased in women. This could reflect that women get cardiovascular disease later than men do, and that treatment in this group both with betablockers and citalopram/escitalopram was considered to be important.

## Conclusion

As people with AD, and especially women, are at high risk for fall-related injuries and syncope, the use of AChEIs should be initiated with caution because of associations with increased rates of bradycardia, syncope, pacemaker insertion, and hip fracture in older adults with dementia (Cronin and Kenny, 2010). Thus, AChEIs should not be a standard treatment of patients with dementia, but decision on prescribing should be weighed on the expected risk profile of each individual, and AChEI therapy should be reconsidered if little or no cognitive improvement is observed early in therapy (Park-Wyllie et al., 2009). Cardiovascular side effects should be monitored, which is specifically important in patients already taking drugs that often cause bradycardia (Mohammad et al., 2017). The use of two or more drugs that prolong the QT interval should be avoided, or if required, monitored closely. Betablockers should not be prescribed in patients with long QT or during treatment with QT prolonging drugs (Cubeddu, 2016).

In summary, with regard to potential ADRs, even the low prevalence of use of haloperidol is concerning. The high prevalence of use of betablockers may give rise to bradycardia and disorders of AV conduction, and induce vertigo and falls, as well as interactions with other drugs with effects on the heart. Due to the high prevalence of use of citalopram/escitalopram, especially in women, a substantial proportion of the patients are at risk of developing pharmacodynamic interactions, especially when combined with AChEIs and betablockers.

### Strengths and Limitations

Using data from the NorPD, we had no access to clinical diagnoses like depression, congestive heart failure and atrial fibrillation, or adverse effects. However, we aimed to explore the association between the use of AChEI and prescription of drugs with effect on the heart. Data from the NorPD gives us a unique opportunity to study drug use patterns, highlighting changes over time in selected drug groups. The large sample size and the long study period is a strength in our study. In Norway, patients in nursing homes more often use antidementia drugs than people living at home (Fog et al., 2019), and the NorPD do not include patients in nursing homes. AChEIs are mainly prescribed for treatment of AD; however, we did not have information with regard to the clinical rationale for prescriptions including etiological diagnoses, severity of the symptoms, and ADL dependency. Purchased drugs were used as a surrogate for consumed drugs and may cause overestimation. However, medicine adherence in AChEI users is expected to be good, as caregivers usually are responsible for drug management for patients with dementia. We did not study dosage differences between men and women, which could be of relevance due to pharmacokinetic differences between the sexes; however, even small doses of the studied drugs may cause serious ADRs in the study group. Medicine use was quantified up to 4 years prior to AChEI initiation, and as only a 1-year wash-out was used to define initiation, the study population included 494 patients who used AChEIs 2–4 years prior to index date. However, the majority of these (61%) had <4 prescriptions in this period, and we have included the 494 in the analysis. Only persistent users were included, and people who were still alive at 2 years post-initiation. People who developed a cardiovascular issue or had a cardiovascular event (potentially due to co-prescribing of medicines associated with cardiovascular problems) might have discontinued AChEIs before they fulfilled the persistence criteria, or have been more likely to die the two first years after AChEI initiation. This could lead to bias in who is included in the study (e.g., people with lower rates of use of the medicines of interest being excluded). There have been no changes in guidelines related to the drugs being studied since 2013. Hence, the therapy that was valid that time is still valid today.

Decisions about prescribing medicines among patients with dementia may be complicated by conflicting recommendations in prescribing guidelines as indicated in this study. A small number of patients treated with AChEIs were prescribed haloperidol, digoxin/digitoxin, and verapamil, while a substantial proportion were prescribed betablockers and citalopram/escitalopram. Prevalence of use of betablockers was reduced by initiation of AChEIs in both sexes. Up to 6% of the study population were prescribed combinations of betablockers and citalopram/citalopram in addition to AChEIs, a combination increasing over the study period and observed most frequently in women in the oldest age group, being the most sensitive group to ADRs. About 44% of the patients were prescribed a drug in at least one of the studied drug groups during the second year after AChEI initiation. The present findings suggest the need for careful medication review with focus on bradycardic and prolonged QT interval in patients treated with AChEIs as an important risk-reducing intervention.

## Data Availability

The original contributions presented in the study are included in the article/Supplementary Material, further inquiries can be directed to the corresponding author.

## Supplementary Material

Supplementary Table S2 Data. Aggregated data supporting Figures 1–7.
