# Supplementary material for: Use of Drugs With Risk of Heart Rate-Related Problems is Common in Norwegian Dementia Patients Treated With Acetylcholinesterase Inhibitors: A Prevalence Study Based on the Norwegian Prescription Database
Source: Front Pharmacol. 2022 Feb 22;12:791578. doi: 10.3389/fphar.2021.791578 (PMC8902444; doi:10.3389/fphar.2021.791578)
Supplement: Supplementary file 1 [file DataSheet1.zip › Supplementary Materials/Supplementary file 1.DOCX]

Supplementary Figure S1. Bullets: Proportion of men and women, respectively, of the study population who used haloperidol, citalopram/escitalopram, verapamil, betablockers or digoxin/digitoxin in the four years before AChEI initiation and the two years after with 95% confidence intervals. Circles: The corresponding age adjusted proportion in the general population. Dashed vertical lines indicate AChEI initiation. The size of each age group in the study population is given on top

Supplementary Table S1. Proportion (%) with 95% confidence interval of men and women, respectively, of the study population (SP) who filled at least one prescription of the studied drugs the fourth and first year before AChEI initiation and the second year after, and corresponding proportion in the age adjusted general population (GP), and prevalence ratios between SP and GP.

|  |  |  | Men | | | Women | | |
| --- | --- | --- | --- | --- | --- | --- | --- | --- |
|  | Age | Population | 4th yr before | 1st yr before | 2nd yr after | 4th yr before | 1st yr before | 2nd yr after |
| Haloperidol | 37-80 | SP | 0.17 (0.07-0.40) | 1.00 (0.70-1.43) | 1.44 (1.07-1.95) | 0.19 (0.10-0.37) | 0.80 (0.57-1.12) | 1.58 (1.24-2.00) |
|  |  | GP | 0.23 (0.22-0.24) | 0.27 (0.26-0.28) | 0.29 (0.28-0.29) | 0.22 (0.21-0.22) | 0.25 (0.24-0.26) | 0.26 (0.25-0.27) |
|  |  | **SP/GP** | **0.75 (0.32-1.75)** | **3.8 (2.61-5.4)** | **5.1 (3.7-6.8)** | **0.87 (0.44-1.72)** | **3.2 (2.30-4.5)** | **6.1 (4.8-7.8)** |
|  | 81-88 | SP | 0.14 (0.04-0.50) | 1.09 (0.67-1.76) | 1.63 (1.10-2.42) | 0.19 (0.09-0.42) | 1.28 (0.94-1.73) | 1.44 (1.07-1.92) |
|  |  | GP | 0.50 (0.45-0.55) | 0.57 (0.51-0.63) | 0.57 (0.51-0.63) | 0.47 (0.43-0.51) | 0.52 (0.48-0.57) | 0.54 (0.50-0.59) |
|  |  | **SP/GP** | **0.27 (0.07-0.99)** | **1.92 (1.17-3.1)** | **2.89 (1.92-4.3)** | **0.41 (0.19-0.89)** | **2.44 (1.77-3.3)** | **2.64 (1.95-3.6)** |
|  | 37-88 | SP | 0.16 (0.08-0.33) | 1.03 (0.77-1.37) | 1.51 (1.19-1.91) | 0.19 (0.11-0.32) | 1.00 (0.80-1.26) | 1.52 (1.26-1.82) |
|  |  | GP | 0.32 (0.31-0.33) | 0.37 (0.36-0.38) | 0.38 (0.37-0.39) | 0.32 (0.31-0.33) | 0.36 (0.35-0.37) | 0.38 (0.37-0.39) |
|  |  | **SP/GP** | **0.50 (0.24-1.03)** | **2.80 (2.09-3.7)** | **4.0 (3.1-5.1)** | **0.59 (0.35-0.99)** | **2.75 (2.19-3.5)** | **4.0 (3.3-4.8)** |
| Citalopram/  Escitalopram | 37-80 | SP | 4.5 ( 3.8- 5.3) | 11.1 (10.0-12.3) | 15.4 (14.1-16.7) | 8.3 ( 7.5- 9.1) | 18.4 (17.3-19.6) | 21.0 (19.8-22.2) |
|  |  | GP | 2.62 (2.59-2.65) | 3.0 (3.00-3.1) | 3.2 (3.1-3.2) | 4.9 (4.9-5.0) | 5.7 (5.7-5.8) | 6.0 (6.0-6.1) |
|  |  | **SP/GP** | **1.72 (1.45-2.03)** | **3.7 (3.3-4.0)** | **4.8 (4.4-5.3)** | **1.68 (1.52-1.85)** | **3.2 (3.0-3.4)** | **3.5 (3.3-3.7)** |
|  | 81-88 | SP | 3.00 ( 2.24- 4.0) | 10.3 ( 8.8-11.9) | 12.7 (11.1-14.5) | 7.4 ( 6.5- 8.3) | 15.0 (13.8-16.3) | 18.4 (17.1-19.8) |
|  |  | GP | 3.6 (3.5-3.8) | 4.2 (4.0-4.3) | 4.3 (4.1-4.5) | 6.2 (6.1-6.3) | 7.1 (7.0-7.3) | 7.3 (7.2-7.5) |
|  |  | **SP/GP** | **0.83 (0.62-1.10)** | **2.46 (2.11-2.87)** | **2.96 (2.57-3.4)** | **1.19 (1.05-1.35)** | **2.11 (1.93-2.29)** | **2.51 (2.32-2.71)** |
|  | 37-88 | SP | 4.0 ( 3.5- 4.6) | 10.8 ( 9.9-11.8) | 14.5 (13.5-15.6) | 7.9 ( 7.3- 8.5) | 17.0 (16.1-17.9) | 19.9 (19.0-20.8) |
|  |  | GP | 2.96 (2.93-2.99) | 3.4 (3.4-3.4) | 3.5 (3.5-3.6) | 5.5 (5.4-5.5) | 6.3 (6.3-6.4) | 6.6 (6.5-6.6) |
|  |  | **SP/GP** | **1.35 (1.17-1.56)** | **3.2 (2.90-3.4)** | **4.1 (3.8-4.4)** | **1.44 (1.33-1.56)** | **2.69 (2.55-2.83)** | **3.0 (2.88-3.2)** |

Supp. Table S1 cont.

|  |  |  | Men | | | Women | | | |
| --- | --- | --- | --- | --- | --- | --- | --- | --- | --- |
|  | Age | Population | 4th yr before | 1st yr before | 2nd yr after | 4th yr before | 1st yr before | | 2nd yr after |
| Verapamil | 37-80 | SP | 1.41 (1.04-1.91) | 1.34 (0.98-1.83) | 1.00 (0.70-1.43) | 1.32 (1.02-1.71) | 1.13 (0.85-1.49) | 1.06 (0.79-1.41) | |
|  |  | GP | 1.40 (1.38-1.42) | 1.34 (1.32-1.36) | 1.27 (1.25-1.29) | 1.37 (1.35-1.39) | 1.39 (1.37-1.41) | 1.36 (1.34-1.38) | |
|  |  | **SP/GP** | **1.01 (0.74-1.36)** | **1.00 (0.73-1.36)** | **0.79 (0.55-1.13)** | **0.96 (0.74-1.25)** | **0.81 (0.61-1.07)** | **0.78 (0.58-1.04)** | |
|  | 81-88 | SP | 2.04 (1.43-2.90) | 1.77 (1.21-2.58) | 1.70 (1.16-2.50) | 1.95 (1.52-2.49) | 1.85 (1.43-2.38) | 1.85 (1.43-2.38) | |
|  |  | GP | 2.43 (2.33-2.53) | 2.08 (1.98-2.19) | 1.79 (1.68-1.90) | 2.64 (2.55-2.73) | 2.37 (2.28-2.47) | 2.14 (2.05-2.24) | |
|  |  | **SP/GP** | **0.84 (0.59-1.20)** | **0.85 (0.58-1.24)** | **0.95 (0.64-1.40)** | **0.74 (0.57-0.95)** | **0.78 (0.60-1.01)** | **0.86 (0.67-1.12)** | |
|  | 37-88 | SP | 1.62 (1.29-2.04) | 1.48 (1.17-1.89) | 1.23 (0.95-1.61) | 1.58 (1.32-1.90) | 1.44 (1.19-1.73) | 1.39 (1.15-1.69) | |
|  |  | GP | 1.74 (1.72-1.77) | 1.59 (1.57-1.61) | 1.44 (1.42-1.46) | 1.90 (1.88-1.92) | 1.80 (1.78-1.83) | 1.69 (1.67-1.71) | |
|  |  | **SP/GP** | **0.93 (0.74-1.17)** | **0.93 (0.73-1.19)** | **0.86 (0.66-1.12)** | **0.83 (0.70-1.00)** | **0.80 (0.66-0.96)** | **0.83 (0.68-1.00)** | |
| Betablockers | 37-80 | SP | 26.5 (24.9-28.1) | 28.8 (27.2-30.5) | 27.9 (26.3-29.6) | 21.2 (20.0-22.5) | 24.2 (23.0-25.5) | 23.2 (22.0-24.5) | |
|  |  | GP | 26.3 (26.2-26.4) | 30.1 (30.0-30.2) | 32.2 (32.1-32.2) | 21.9 (21.9-22.0) | 25.3 (25.2-25.4) | 27.2 (27.1-27.3) | |
|  |  | **SP/GP** | **1.01 (0.95-1.07)** | **0.96 (0.90-1.01)** | **0.87 (0.82-0.92)** | **0.97 (0.91-1.03)** | **0.96 (0.91-1.01)** | **0.85 (0.81-0.90)** | |
|  | 81-88 | SP | 32.5 (30.2-35.0) | 36.6 (34.1-39.1) | 35.3 (32.9-37.7) | 29.5 (27.9-31.1) | 33.6 (32.0-35.3) | 31.8 (30.2-33.5) | |
|  |  | GP | 35.4 (35.1-35.8) | 38.3 (37.9-38.6) | 39.3 (38.9-39.7) | 31.6 (31.4-31.9) | 34.7 (34.4-35.0) | 35.8 (35.5-36.1) | |
|  |  | **SP/GP** | **0.92 (0.85-0.99)** | **0.96 (0.89-1.02)** | **0.90 (0.84-0.96)** | **0.93 (0.88-0.98)** | **0.97 (0.92-1.02)** | **0.89 (0.84-0.94)** | |
|  | 37-88 | SP | 28.5 (27.2-29.9) | 31.4 (30.0-32.8) | 30.4 (29.1-31.8) | 24.7 (23.8-25.7) | 28.2 (27.2-29.2) | 26.9 (25.9-27.9) | |
|  |  | GP | 29.4 (29.3-29.5) | 32.9 (32.8-32.9) | 34.6 (34.5-34.7) | 26.1 (26.0-26.2) | 29.3 (29.2-29.4) | 30.9 (30.8-30.9) | |
|  |  | **SP/GP** | **0.97 (0.93-1.02)** | **0.96 (0.91-1.00)** | **0.88 (0.84-0.92)** | **0.95 (0.91-0.99)** | **0.96 (0.93-1.00)** | **0.87 (0.84-0.90)** | |

Supp. Table S1 cont.

|  |  |  | Men | | | Women | | | |
| --- | --- | --- | --- | --- | --- | --- | --- | --- | --- |
|  | Age | Population | 4th yr before | 1st yr before | 2nd yr after | 4th yr before | 1st yr before | | 2nd yr after |
| Digoxin/  Digitoxin | 37-80 | SP | 2.23 (1.76-2.84) | 2.96 (2.40-3.6) | 2.82 (2.28-3.5) | 1.22 (0.93-1.60) | 1.65 (1.31-2.07) | 1.62 (1.28-2.05) | |
|  |  | GP | 2.24 (2.21-2.26) | 2.51 (2.48-2.54) | 2.44 (2.42-2.47) | 1.25 (1.23-1.27) | 1.57 (1.55-1.59) | 1.65 (1.63-1.68) | |
|  |  | **SP/GP** | **1.00 (0.79-1.27)** | **1.18 (0.96-1.45)** | **1.15 (0.93-1.43)** | **0.98 (0.75-1.28)** | **1.05 (0.83-1.32)** | **0.98 (0.78-1.24)** | |
|  | 81-88 | SP | 4.3 (3.4-5.4) | 5.5 (4.5-6.8) | 5.2 (4.2-6.5) | 2.36 (1.89-2.95) | 3.7 (3.1-4.4) | 3.6 (3.0-4.3) | |
|  |  | GP | 5.6 (5.4-5.7) | 5.5 (5.3-5.7) | 4.9 (4.7-5.1) | 4.2 (4.1-4.3) | 4.6 (4.5-4.7) | 4.3 (4.2-4.5) | |
|  |  | **SP/GP** | **0.77 (0.60-0.98)** | **1.00 (0.81-1.24)** | **1.08 (0.86-1.34)** | **0.56 (0.45-0.71)** | **0.81 (0.67-0.97)** | **0.83 (0.69-1.00)** | |
|  | 37-88 | SP | 2.92 (2.46-3.5) | 3.8 (3.3-4.4) | 3.6 (3.1-4.2) | 1.71 (1.43-2.03) | 2.52 (2.18-2.90) | 2.46 (2.13-2.84) | |
|  |  | GP | 3.4 (3.3-3.4) | 3.5 (3.5-3.5) | 3.3 (3.2-3.3) | 2.49 (2.46-2.51) | 2.84 (2.81-2.86) | 2.78 (2.75-2.81) | |
|  |  | **SP/GP** | **0.87 (0.74-1.03)** | **1.09 (0.93-1.26)** | **1.12 (0.96-1.30)** | **0.69 (0.58-0.82)** | **0.89 (0.77-1.02)** | **0.89 (0.77-1.02)** | |
| Any of the above | 37-80 | SP | 31.2 (29.6-33.0) | 38.7 (37.0-40.5) | 41.1 (39.3-42.9) | 28.8 (27.5-30.2) | 39.5 (38.0-41.0) | 41.7 (40.2-43.2) | |
|  |  | GP | 29.7 (29.6-29.8) | 33.6 (33.5-33.7) | 35.6 (35.5-35.7) | 26.9 (26.8-27.0) | 30.7 (30.6-30.8) | 32.7 (32.6-32.7) | |
|  |  | **SP/GP** | **1.05 (1.00-1.11)** | **1.15 (1.10-1.21)** | **1.15 (1.10-1.20)** | **1.07 (1.02-1.12)** | **1.29 (1.24-1.33)** | **1.28 (1.23-1.32)** | |
|  | 81-88 | SP | 38.5 (36.0-41.0) | 47.0 (44.5-49.6) | 48.1 (45.5-50.6) | 36.3 (34.6-38.0) | 45.9 (44.1-47.6) | 46.8 (45.1-48.6) | |
|  |  | GP | 41.4 (41.1-41.7) | 43.9 (43.6-44.3) | 44.5 (44.1-44.9) | 38.9 (38.7-39.2) | 41.9 (41.6-42.2) | 42.7 (42.4-43.1) | |
|  |  | **SP/GP** | **0.93 (0.87-0.99)** | **1.07 (1.01-1.13)** | **1.08 (1.02-1.14)** | **0.93 (0.89-0.98)** | **1.09 (1.05-1.14)** | **1.10 (1.05-1.14)** | |
|  | 37-88 | SP | 33.7 (32.3-35.1) | 41.5 (40.1-43.0) | 43.4 (42.0-44.9) | 32.0 (31.0-33.1) | 42.2 (41.1-43.3) | 43.9 (42.7-45.0) | |
|  |  | GP | 33.6 (33.6-33.7) | 37.1 (37.0-37.2) | 38.6 (38.5-38.7) | 32.0 (32.0-32.1) | 35.5 (35.4-35.6) | 36.9 (36.9-37.0) | |
|  |  | **SP/GP** | **1.00 (0.96-1.04)** | **1.12 (1.08-1.16)** | **1.13 (1.09-1.16)** | **1.00 (0.97-1.03)** | **1.19 (1.16-1.22)** | **1.19 (1.16-1.22)** | |
